# Purtscher's like retinopathy - A rare ocular complication of acute pancreatitis

**DOI:** 10.1016/j.ijscr.2024.109881

**Published:** 2024-06-06

**Authors:** Havil Stephen Alexander Bakka, Perumalla Karthik Babu, L.V. Simhachalam Kutikuppala, M.V. Ramana Reddy, Golla Varshitha

**Affiliations:** a Department of General Surgery, Ramesh Sanghamitra Hospitals, Ongole 523001, Andhra Pradesh, India; b Department of General Surgery, Dr NTR University of Health Sciences, Vijayawada, Andhra Pradesh, India; c Department of Ophthalmology, Modern Eye Hospital and Research Centre, Ongole 523001, Andhra Pradesh, India; d Department of Internal Medicine, International School of Medicine (ISM), Bishkek, Kyrgyzstan

**Keywords:** Ocular complication, Pancreatitis, Purtscher's retinopathy

## Abstract

**Introduction and importance:**

Purtscher retinopathy is the rare form of occlusive microvasculopathy, characterized by multiple retinal white areas around the optic nerve head and fovea with paravascular clearing and may be related to intraretinal hemorrhages. Acute Pancreatitis (AP) is one of the most common gastrointestinal reasons for hospital admissions globally. The complications of Acute Pancreatitis may include Purtscher's-like retinopathy, which has a low incidence rate of less than 0.24 instances per million cases. This case report highlights the value of thorough medical history taking and examination, and it apprises the consideration of ophthalmological manifestation in patients of Acute Pancreatitis.

**Case presentation:**

A 34-year-old female came to the emergency room due to intense abdominal pain associated with nausea and vomiting, which worsened over the last 24 h. The pain was described as continuous, sharp, and cramping-like in the upper abdomen, radiating to the back. Lab tests revealed elevated serum amylase and lipase levels, indicating pancreatitis, along with slight leukocytosis. A contrast-enhanced CT scan confirmed acute pancreatitis with mild inflammation and enlargement of the pancreas. Two days after admission, the patient experienced a sudden and painless loss of central vision in both eyes. There was no history of trauma or any other significant relevant history, other than pancreatitis. The ophthalmologist's examination found reduced visual acuity (6/60 in the right eye, 3/60 in the left eye), normal corneas, and anterior chambers.

**Discussion:**

Inkeles and Walsh established the first link between acute pancreatitis and Purtscher-like retinopathy when they reported three cases of the distinctive retinal appearance in individuals with acute pancreatitis in 1975.

**Conclusion:**

The recovery and prognosis in cases of Purtscher-like retinopathy is variable and further research is required to ascertain the usage of corticosteroids and pentoxifylline in improving the course of a patient's with Purtscher's-like retinopathy.

## Highlights

• Purtscher retinopathy is the rare form of occlusive microvasculopathy
• Complications of Acute Pancreatitis may include Purtscher's retinopathy, with low incidence of < 0.24 instances per million.
• This case report highlights the value of thorough medical history taking and examination in patients of Acute Pancreatitis.
• The recovery and prognosis in cases of Purtscher-like retinopathy is variable and further research is required.

## 1 Introduction

Acute Pancreatitis (AP) is one of the most common gastrointestinal reasons for hospital admissions globally. There are many risk factors for acute pancreatitis, but cholelithiasis (gallstones) or chronic alcohol use account for 70–75 % of all cases. The predominant signs and symptoms of AP are nausea, vomiting, and severe abdominal pain. However, as the disease progresses, manifestation from other organs can be possible [1,2]. Purtscher retinopathy is the rare form of occlusive microvasculopathy, characterized by multiple retinal white areas around the optic nerve head and fovea with paravascular clearing and may be related to intraretinal hemorrhages [3]. Otmar Purtscher originally identified Purtscher retinopathy in a man who experienced cranial trauma after falling from a tree in the year 1910 [4]. The retinopathy typically has a favorable outcome, with the ocular lesions disappearing and visual acuity returning within 4–6 weeks in the majority of cases. However, some patients might still have lesions, particularly if there is optical atrophy [5]. Purtscher retinopathy is mostly caused due to the traumatic injury, but the causes of Purtscher's-like retinopathy are not traumatic. Numerous conditions, including antibiotic allergy, myocardial infarction, connective tissue illnesses, renal failure, delivery, and bone marrow transplant, have been linked to its prevalence. Additionally, complications of AP may include Purtscher's-like retinopathy, which has a low incidence rate of less than 0.24 instances per million cases [1,6]. This work has been reported in line with the SCARE criteria [7].

## 2 Case report

A 34-year-old female working as a software engineer, came to the emergency room due to intense abdominal pain associated with nausea and vomiting, which worsened over the last 24 h. The pain was described as continuous, sharp, and cramping-like in the upper abdomen, radiating to the back. The pain is aggravated by eating and lying back and relieved by leaning forward. She does not consume alcohol or tobacco, but her diet consisted of fried and spicy foods, and irregular meal patterns due to her demanding job. Her medical history revealed occasional indigestion and bloating. She is married and has a 3-year-old child with an uneventful delivery.

Upon examination, her vital signs were normal, with a heart rate of 88 bpm, blood pressure of 120/80 mmHg, and no fever. An abdominal evaluation revealed tenderness in the epigastric region without guarding, rigidity, or rebound tenderness. Bowel sounds were present. Lab tests revealed elevated serum amylase and lipase levels, indicating pancreatitis, along with slight leukocytosis. Other blood parameters were normal. Abdominal ultrasound displayed swollen pancreas but no gallstones or bile duct blockages. A contrast-enhanced CT scan confirmed acute pancreatitis with mild inflammation and enlargement of the pancreas.

**Fig. 1:**
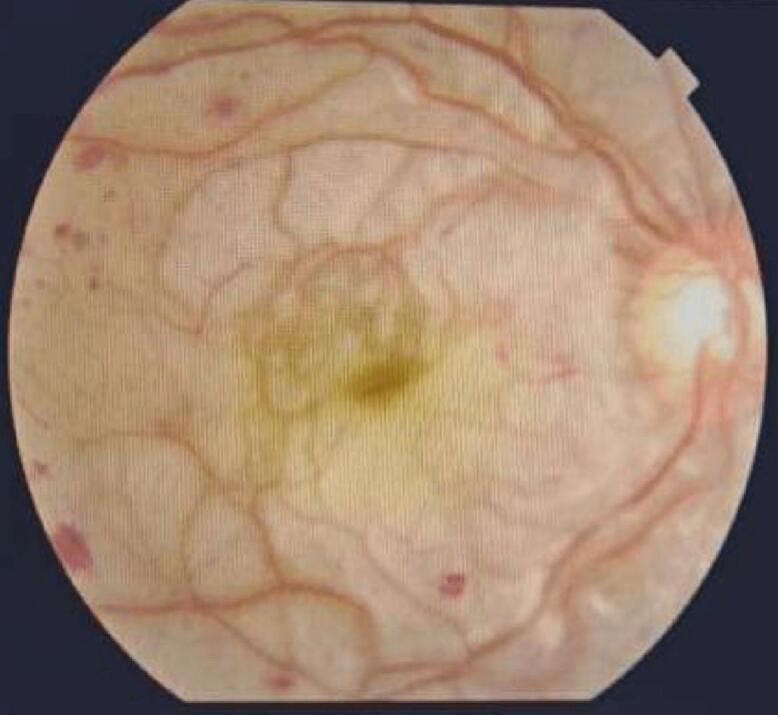
Right Eye fundus: Cotton wool spots, Superficial Hemorrhages, Macula and Foveal edema.

**Fig. 2:**
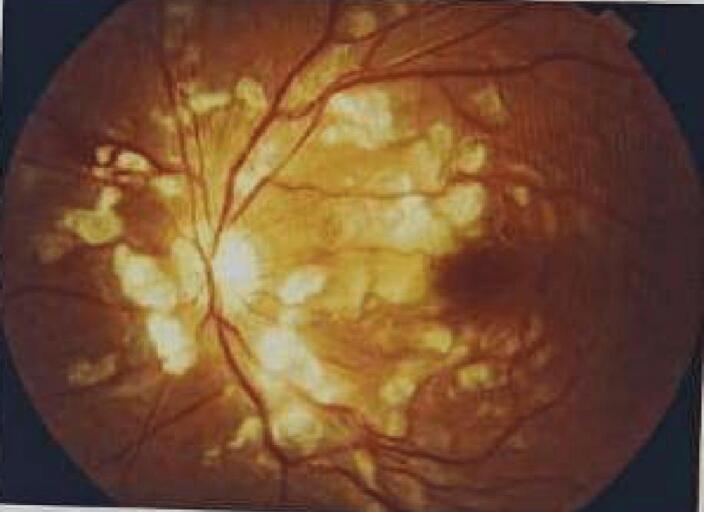
Left Eye Fundus: Cotton wool spots, Superficial Hemorrhages, Macula and Foveal edema.

**Fig. 3:**
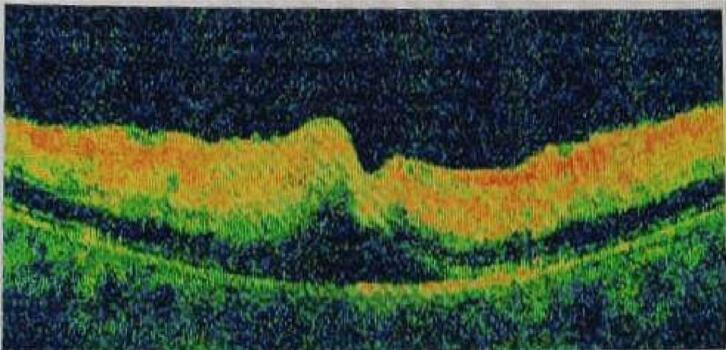
Ocular coherence tomography (OCT) of the left eye horizontally cut at the level of the fovea showing inner retinal edema with alteration of the foveal region and subretinal fluid in the subfoveal region.

Two days after admission, the patient experienced a sudden and painless loss of central vision in both eyes. There was no history of trauma or any other significant relevant history, other than pancreatitis. The ophthalmologist's examination found reduced visual acuity (6/60 in the right eye, 3/60 in the left eye), normal corneas, and anterior chambers. Pupils were sluggish in reacting to light, and lenses were clear in both eyes. Intraocular pressures were within normal limits (NCT 15 mmHg and 12 mmHg in both eyes, respectively). Fundus examination displayed whitish patches (cotton wool spots), superficial hemorrhages, and macular oedema, with no cells in the vitreous (Fig. 1, Fig. 2). Optical coherence tomography (OCT) revealed thickening of the fovea and macula due to oedema (Fig. 3), consistent with Purtscher-like retinopathy. With treatment, pancreatitis resolved, and the patient's condition improved using sub-tenon steroids (40 mg triamcinolone acetonide), followed by a repeat injection three months later. Four months later, her vision had improved to 20/20 in both eyes during an ophthalmic evaluation, and no visual issues were noted as the condition progressed.

## 3 Discussion

When Inkeles and Walsh reported three cases of the distinctive retinal appearance in individuals with acute pancreatitis in 1975, they established the first link between acute pancreatitis and Purtscher-like retinopathy [8]. However, less than 2 % of Acute pancreatitis patients experienced abrupt visual loss linked to retinal abnormalities such as retinal hemorrhages and oedema. The precise mechanisms responsible for its occurrence remain unidentified [1,4]. Retinal hemorrhages and ischaemia are linked to Purtscher-like retinopathy, which is most likely caused by the complement-mediated leukoembolization. Although the precise mechanism underlying pancreatic damage-induced release of proteolytic enzymes into the systemic circulation is unclear, it has been hypothesized that complement cascade activation, C5a-induced platelet, leukocyte, and fibrin aggregate formation can result in retinal embolization and ischemia [8,9]. Fundus fluorescein angiography (FFA) can be done to assess the amount of vascular perfusion and Optical coherence tomography (OCT) can help in evaluation of the macular edema resulting from this condition. Visual prognosis is usually bad in these cases, where the degree of visual impairments varies in resolution. In half of the cases, spontaneous improvement of over two Snellen lines can be expected [3,10]. As in the previous reported cases, although there was some spontaneous visual recovery in this case, it was likely incomplete because of ischemia alterations occurred at the macula in due course of the illness. Since Purtscher-like retinopathy can cause extremely disabling visual symptoms, more research is necessary to create more potent treatment options. Increasing awareness among healthcare professionals about the possibility of this rare complication can facilitate in prompt ophthalmological evaluation in cases of acute pancreatitis [2].

## 4 Conclusion

The recovery and prognosis in cases of Purtscher-like retinopathy is variable and further research is required to ascertain the usage of corticosteroids and pentoxifylline in improving the course of a patient's with Purtscher's-like retinopathy. This case report highlights the value of thorough medical history taking and examination, and it apprises the consideration of ophthalmological manifestation in patients of Acute Pancreatitis.

Informed consent was taken from the patient for publication of the case report.

## Patient consent

Written informed consent was obtained from the patient for publication and any accompanying images. A copy of the written consent is available for review by the Editor-in-Chief of this journal on request.

## Ethical approval

Ethics approval is not required for case reports or case series deemed not to constitute research at our institution.

## Funding

None to disclose.

## Author contribution

Dr. Havil Stephen Alexander Bakka – Concept, Review of Literature, Writing and Editing the paper.

Dr. Perumalla Karthik Babu - Concept, Writing and Editing the paper.

Dr. L V Simhachalam Kutikuppala - Concept, Writing and Editing the paper.

Dr. M V Ramana Reddy – Writing, Editing and Submitting the paper.

Dr. Varshitha G - Writing and Editing the paper.

## Guarantor

Varshitha Golla.

## Research registration number

Not Applicable.

## Funding acknowledgements

None to disclose.

## Conflict of interest statement

None to disclose.
